# Repercussions of the COVID-19 pandemic on the epidemiology of facial fractures: a retrospective cohort study

**DOI:** 10.1007/s10006-023-01200-3

**Published:** 2023-12-07

**Authors:** Artur de Sousa Lima, João Victor Frazão Câmara, Josué Junior Araujo Pierote, Lethycia Almeida Santos, Carolina Ruis Ferrari, Maria Cândida de Almeida Lopes, Ana Cristina Vasconcelos Fialho

**Affiliations:** 1 Department of Pathology and Dental Clinic, Health Sciences Center, Federal University of Piauí, Campus Universitário Ministro Petrônio Portella, Teresina, PI 64049-550 Brazil; 2 Clinic of Operative Dentistry, Periodontology and Preventive Dentistry, Saarland University Hospital, Saarland University, Kirrberger Str. 100, Building 73, 66421 Homburg, Saar Germany; 3 Santo Amaro University, Rua Isabel Schmidt 349, São Paulo, SP 04743-030 Brazil; 4 Department of Biological Sciences, Bauru School of Dentistry, University of São Paulo, Bauru, SP Brazil

## Introduction

Trauma is among the main causes of death and morbidity in the world, according to data from the World Health Organization, and is considered a public health problem [1, 2]. Injuries to the head and face may be responsible for 50% of all traumatic deaths when associated with other injuries such as cervical, abdominal, thoracic, and brain trauma according to more recent research [3–5].

Facial trauma can generate devastating sequelae, mainly due to the deformities and scars caused on the face, which is the most visible and exposed part of the human body, often leading the individual to develop psychological problems such as depression, anxiety, and stress, as the severity of the injuries increases [6–8]. This severity is usually accentuated when associated with dental trauma, bones, and soft tissues, in addition to regions such as the brain, eyes, ears, chest, and abdomen [9]. Facial trauma remains one of the leading causes of morbidity and mortality, with large costs for national healthcare systems because they often necessitate intensive care unit stays as a result of polytrauma [10]. Such sequelae can generate months of hospitalization and surgical and rehabilitation procedures, thus requiring a longer time away from work activities, increased financial costs, and a great socioeconomic impact on society, negatively affecting the quality of life [11].

Severe Acute Respiratory Syndrome (SARS-CoV-2) is an infectious disease caused by COVID-19 which has negatively impacted the health system, causing changes in the epidemiological pattern of facial fractures, mainly in the incidence and etiology [12]. The COVID-19 pandemic led to the adoption of social restriction measures to isolate the community to mitigate the spread of coronavirus and differ according to the geographic area they occur. In Brazil, the closure of educational facilities and non-essential services were the measures applied throughout the national territory [13]. Specifically, maxillofacial procedures around the upper aerodigestive tract became a challenge due to the risk of aerosolization and the spread of the COVID-19 virus [14]. Therefore, conservative treatment for maxillofacial trauma was recommended to minimize the aerosolization risk. In addition, delayed treatment of nasal bone fractures was recommended to minimize the high risk of aerosolization associated with nasal cavity instrumentation [15–17].

Moreover, the treatment of facial fractures is complex and requires an appropriate multidisciplinary approach and intervention, specifically in the COVID-19 pandemic. Despite these recommendations, there has not been a nationally standardized approach to facial trauma management during the COVID-19 pandemic; therefore, the impact of COVID-19 on trauma management remains unclear. Therefore, the aim of this study was to identify the repercussions of social distancing during COVID-19 on the epidemiology of facial fractures in the capital of Brazil.

## Methodology

### Ethical aspects

The research was ethically developed according to the Declaration of Helsinki (World Medical Association) and the criteria in Resolution 466/12 of the National Research Ethics Commission (CONEP) of the National Health Council (CNS), which regulates the ethical guidelines on researches with human beings. This study was approved by the Ethics and Research Committee from the Federal University of Piauí—UFPI, under opinion number 4.630.301 issued in 04/05/2021.

### Study characterization

The study provides an observational retrospective cohort study to identify the impact of social distancing during the COVID-19 pandemic on the epidemiology of types of facial fractures in the capital of Brazil, with a period before and during social isolation. The research was based on STROBE guidelines for observational studies.

### Survey sample

The sample consists of all medical records of patients with facial fractures assisted by the Oral and Maxillofacial Surgery and Traumatology Service of the Hospital de Urgência de Teresina—HUT in Piauí, in 2019 and 2020, which attended to inclusion criteria. The records were selected through the International Classification of Diseases (ICD-10) and the year the fracture occurred.

### Data source

Information was collected from medical records of the Hospital de Urgência de Teresina—HUT from March to December 2019 (2019 cohort) and from March to December 2020 (period of social distancing caused by the COVID-19 pandemic–2020 cohort). HUT was chosen because it is a referral public hospital in the region in cases of urgency care and emergency of medium and high complexity.

### Study variables

The primary variable occurred when the subject was diagnosed with a facial fracture. The 2019 cohort included data collected in 2019 (no social distancing policies) and the 2020 cohort (period of social distancing by COVID-19).

Outcome variables were fracture type (nasal bones fracture (S02.2), orbital floor fracture (S02.3), zygomatic and maxillary bones fracture (S02.4), mandible fracture (S02.6), and multiple fractures involving skull and face bones (S02.7) organized according to International Classification of Diseases (ICD), etiology of fractures (physical aggression, motorcycle and car accidents, gunshot, bladed weapon, bicycle, falls, others and shock/object/person), and severity of injury.

Comprehensive Facial Injury (CFI) [18] was used to calculate the severity of facial injury. A CIF is represented as a numerical value as the sum of all facial injuries, and the result indicates the severity. The CFI divides the facial region into three horizontal thirds: the lower third (including the symphysis, body, angles, vertical branches and condyles, lower dentoalveolar region), the middle third (upper jaw, upper dentoalveolar arch, zygoma, lateral and medial walls, floor of the orbits and nasal bones), and the upper third (orbital roof, frontal bone). After obtaining the values corresponding to the injury severity, the sum result was categorized in CFI from 0 to 1—mild, 2 to 4—moderate, and 5 to 15—severe. The variables: house location (rural and urban), sex, therapy, and age were included in the study.

### Inclusion and exclusion criteria

The inclusion criteria considered the hospitalized patients, during the determined period, with subsequent analysis of the records to identify the types of facial fractures. Incomplete or blank medical records were excluded; isolated tooth and old/chronic maxillofacial fractures and patients from other cities were not considered for the analysis, as well as the ones who were not within the inclusion criteria.

### Statistical analysis

The data obtained were tabulated in the Microsoft Office Excel 2019 application. Subsequently, they were stored in SPSS software version 22.0 (SPSS, Chicago, IL, EUA), with a minimum significance level of 5% and were presented in tables and graphs.

A descriptive analysis of the data was performed, calculating the frequency and percentage for the categorical variables, while for the quantitative ones, the average and standard deviation were calculated. After applying the Kolmogorov-Smirnov test for normality and homogeneity, the data revealed a non-normal distribution (*p*<0.05), and non-parametric tests were used to analyze the differences among the variables. The non-parametric tests applied were chi-square and Fisher exact when it was not possible to use the chi-square test.

## Results

### Sex and age

A total of 406 medical records of patients with facial fractures were analyzed between 2019 and 2020, of which 279 records corresponded to 2019 and 127 to 2020. The results indicated a reduction of 54.5% of the frequency of facial fractures during the global crisis caused by COVID-19.

Males were more affected compared to females in both cohorts. In 2019, 279 fractures were identified being the age group between 19 and 29 years most affected (*n*=86; 30.82%; *n*=16; 5.73%), followed by the age group from 30 to 39. In 2020, 127 fractures were identified, and the most affected age groups were 30–39 years old and 40–49 years for both sexes (Table 1).

**Table 1:** Distribution of percentage and frequency of facial fractures by age, year, and sex in Hospital de Urgência de Teresina - Teresina, Piauí, Brazil – 2019 and 2020

Age	2019 cohort			2020 cohort		
	M	F	Total	M	F	Total
1 to 18	21	4	25	9	1	10
	8.9%	9.1%	9.0%	8.7%	4.3%	7.9%
19 to 29	86	19	105	36	5	41
	36.6%	43.2%	37.6%	34.6%	21.7%	32.3%
30 to 39	57	13	70	24	10	34
	24.3%	29.5	25.1%	23.1%	43.5%	26.8%
40 to 49	37	6	43	20	5	25
	15.7%	13.6%	15.4%	19.2%	21.7%	19.7%
50 to 100	34	2	36	15	2	17
	14.5%	4.5%	12.9%	14.4%	8.7%	13.4%
Total	235	44	279	104	23	127
	84.2	15.8%	100%	81.19%	18.1%	100%

*M*, male; *F*, female

Source: Data obtained from medical records in Hospital de Urgência de Teresina – PI, 2019 and 2020

The average age of the patients in the 2019 and 2020 cohorts were 33.95 and 34.99 years old, respectively, being 34.28 the general average. Bivariate inferential statistics using the Pearson chi-square test in relation to year, age, and sex variables, in the 2019 and 2020 cohorts, presented a value of *p*>0.05, demonstrating that there is no statistically significant relationship.

### Place of residence

Demographic data in both years (2019 and 2020) indicated the urban zone as the place where most facial fractures occurred, with a frequency of 88.2% compared to the rural zone.

### Type of fracture

A total of 406 fractures were identified (2019 and 2020), of which the most frequent involved the zygomatic/maxillary bone 46.1%, followed by fractures of the mandible (27.3%) and nasal bones (23.9%). Table 2 shows the distribution of fractures in 2019 and 2020.

**Table 2:** Distribution of percentages and frequencies of types of facial fractures according to the year of occurrence (2019 and 2020) and etiological factor, Hospital de Urgência de Teresina - Teresina, Piauí, Brazil – 2019 and 2020

Etiology	Type of fracture																							
	*Nasal bones fracture^1^				Orbital floor fracture				*Zygomatic and maxillary bone fracture^1^				Mandible fracture				Multiple fractures involving face and skull bones				Total			
	2019		2020		2019		2020		2019		2020		2019		2020		2019		2020		2019		2020	
	*n*	%	*n*	%	*n*	%	*n*	%	*n*	%	*n*	%	*n*	%	*n*	%	*n*	%	*n*	%	*n*	%	*n*	%
^1^Physical aggression*	13	35.14%	***12***	***63.16%***^***4***^	0	0.00%	0	0.00%	***10***	***27.03%***^***2***^	6	31.58%	13	35.14%	1	5.26%	1	2.70%	0	0.00%	37	100%	19	100%
^1^Motorcycle accident*	16	14.55%	2	3.70%	0	0.00%	0	0.00%	***64***	***58.18%*** ^***3***^	***33***	***61.11%*** ^***6***^	37	33.64%	18	33.33%	2	1.82%	1	1.85%	110	100%	54	100%
Car a ccident	1	11.11%	0	0.00%	1	11.11%	0	0.00%	4	44.44%	0	0.00%	110	1222.22%	0	0.00%	0	0.00%	0	0.00%	9	100%	0	100%
Gunshot	0	0.00%	0	0.00%	0	0.00%	0	0.00%	7	63.64%	0	0.00%	9	81.82%	1	50.00%	1	9.09%	1	50.00%	11	100%	2	100%
Bladed weapon	4	44.44%	2	50.00%	0	0.00%	0	0.00%	4	44.44%	1	25.00%	11	122.22%	1	25.00%	0	0.00%	0	0.00%	9	100%	4	100%
Bicycle	0	0.00%	2	28.57%	1	10.00%	0	0.00%	6	60.00%	4	57.14%	9	90.00%	1	14.29%	0	0.00%	0	0.00%	10	100%	7	100%
Falls	11	26.19%	3	17.65%	0	0.00%	0	0.00%	18	42.86%	7	41.18%	10	23.81%	7	41.18%	1	2.38%	0	0.00%	42	100%	17	100%
Others	10	37.04%	7	38.89%	0	0.00%	0	0.00%	8	29.63%	4	22.22%	42	155.56%	7	38.89%	1	3.70%	0	0.00%	27	100%	18	100%
Shock/object/person	9	37.50%	***5***	***83.33%*** ^***5***^	0	0.00%	0	0.00%	11	45.83%	0	0.00%	27	112.50%	1	16.67%	1	4.17%	0	0.00%	24	100%	6	100%
Total	64	22.94%	33	25.98%	2	0.72%	0	0.00%	132	47.31%	55	43.31%	37	26.52%	37	29.13%	24	8.60%	2	1.57	279	100%	127	100%

^1^Pearson chi-square test; *n* = frequency; % percentage

*Level of significance 5%; values of *p* – CI 95% (^2^*p*=0.006; ^3^*p*=0.003; ^4^*p*=0.000; ^5^*p*=0.000; ^6^*p*=0.000)

Source: Data obtained from the medical records in Hospital de Urgência de Teresina – PI, 2019 and 2020

Pearson’s chi-square inferential statistical test showed that there is a significant association between the type of fracture and etiological factor in both years. In Table 2, it is possible to observe the descriptive data and pairwise comparisons using statistical tests, in which the application of post-hoc analyses was carried out to identify the exact comparisons that present statistical significance. In the 2019 and 2020 cohort, a statistically significant proportion of fractures of the zygomatic bone and nasal bones caused mainly by physical aggression was observed [(*x*² (32) = 61.360; *p*=0.006; 2019) / (*x*² (21) =79, 13; *p=*0.000; 2020)]. Furthermore, fractures caused by shock/object/person [(*x*² (21) =79.13; *p*=0.000] in 2020 mainly caused fractures of the nasal bones. Thus, the data in Table 2 demonstrates that victims of facial fractures caused by aggression and shock have a greater predisposition to fracture the nasal bone. Also, in 2020 and 2019, motorcycle accidents that caused fractures of the zygomatic/maxillary bone presented a significantly higher proportion of cases [(*x*² (32) = 61.360; *p*= 0.003; 2019) / (*x*² (21) =79.130; *p*=0.000; 2020)], showing that motorcyclists have a greater predisposition to fracture the zygomaticus when compared to other etiological factors (Table 2).

### Etiology

Motorcycle accidents (39.4%), falls (15.1%), and physical aggression (13.3%) were the most common causes of facial fracture in 2019. The etiological pattern varied in different ages, and the most frequent were motorcycle accidents—age 19–29 (15.77%) and age 30–39 (9.67%) in 2019. In 2020, motorcycle accidents continued to be more frequent (*n*=41; 32.28%), but the most affected age group was 30–39 (Table 3).

**Table 3:** Etiology and distribution of age of facial fractures in 2019 and 2020 cohorts with their respective frequencies and percentages, Hospital de Urgência de Teresina - Teresina, Piauí, Brazil – 2019 and 2020

Etiology	Age																							
	1 a 18				20 a 29				*30 a 39^1^				40 a 49				*50 a 100^1^				Total			
	2019		2020		2019		2020		2019		2020		2019		2020		2019		2020		2019		2020	
	*N*	%	*n*	%	*n*	%	*n*	%	*n*	%	*N*	%	*n*	%	*n*	%	*n*	%	*n*	%	*n*	%	*n*	%
^1^Physical aggression*	1	2.70%	0	0.00%	10	27.03%	6	31.58%	***20***	***54.05%*** ^***2***^	5	26.32%	3	8.11%	5	26.32%	3	8.11%	3	15.79%	37	100%	19	100%
Motorcycle accident	10	9.09%	6	11.11%	44	40.00%	16	29.63%	27	24.55%	20	37.04%	20	18.18%	9	16.67%	9	8.18%	3	5.56%	110	100%	54	100%
Car accident	0	0.00%	0	0.00%	5	55.56%	0	0.00%	0	0.00%	0	0.00%	1	11.11%	0	0.00%	3	33.33%	0	0.00%	9	100%	0	100%
Gunshot	2	18.18%	0	0.00%	8	72.73%	2	100.00%	0	0.00%	0	0.00%	1	9.09%	0	0.00%	0	0.00%	0	0.00%	11	100%	2	100%
Bladed weapon	0	0.00%	0	0.00%	3	33.33%	1	25.00%	3	33.33%	1	25.00%	3	33.33%	2	50.00%	0	0.00%	0	0.00%	9	100%	4	100%
Bicycle	0	0.00%	0	0.00%	4	40.00%	4	57.14%	2	20.00%	2	28.57%	2	20.00%	0	0.00%	2	20.00%	1	14.29%	10	100%	7	100%
^1^Falls*	6	14.29%	2	11.76%	12	28.57%	3	17.65%	1	2.38%	3	17.65%	8	19.05%	2	11.76%	**15**	**35.71%** ^**3**^	7	41.18%	42	100%	17	100%
Others	3	11.11%	1	5.56%	8	29.63%	7	38.89%	8	29.63%	2	11.11%	4	14.81%	5	27.78%	4	14.81%	3	16.67%	27	100%	18	100%
Shock/object/person	3	12.50%	1	16.67%	11	45.83%	2	33.33%	9	37.50%	1	16.67%	1	4.17%	2	33.33%	0	0.00%	0	0.00%	24	100%	6	100%
Total	25	8.96%	10	7.87%	105	37.63%	41	32.28%	70	25.09%	34	26.77	4	15.41%	25	19.69%	36	12.90%	17	13.39%	279	100%	127	100%

^1^Pearson chi-square test; *n* = frequency; % percentage

*Level of significance 5%; values of *p* – CI 95% - (^2^*p*=0.000; ^3^*p*=0.000); 2020 = *p*>0.05

Source: Data obtained from the medical records in Hospital de Urgência de Teresina – PI, 2019 and 2020

Total cross-tabulations related to year and age are observed in Table 3. The etiology analysis related to sex showed that motorcycle accidents were the main cause of fractures for males and females in 2019 (Table 4). Falls (14.9%) and physical aggression (11.5%) were the second and third most frequent causes, respectively, more frequent for males, but physical aggression (22.7%) and falls (15.9%) were the second and third most frequent causes for females in 2019.

**Table 4:** Distribution pattern of etiological factor related to sex, with percentage and frequency in 2019 and 2020, Hospital de Urgência de Teresina - Teresina, Piauí, Brazil – 2019 and 2020

Etiology		Sex (2019)		Total	Value of *p*	Sex (2020)		Total	Value of *p*
		M [*n* (%)]	F [*n* (%)]		Fisher exact	M [*n* (%)]	F [*n* (%)]		Exato de Fisher
	Physical aggression	**27 (11.5)**	**10 (22.7)**	37 (13.3)	(*x*² (8) = 5.74; *p*=0.659)	**17 (16)**	**2 (9)**	19 (15)	(*x*² (7) = 6.196; *p*=0.454)
	Motorcycle accident	**94 (40.0)**	**16 (36.4)**	110 (39.3)		**42(40)**	**12 (52)**	54 (43)	
	Car accident	8 (3.4)	1 (2.3)	9 (3.2)		0 (0)	0 (0)	0 (0)	
	Gunshot	10(4.3)	1 (2.3)	11 (3.9)		2 (2)	0 (0)	2 (2)	
	Bladed weapon	7 (3.0)	2 (4.5)	9 (3.2)		3 (3)	1 (4)	4 (3)	
	Bicycle	10 (4.3)	0 (0)	10 (3.6)		5 (5)	2 (9)	7 (6)	
	Falls	**35 (14.9)**	**7 (15.9)**	42 (15.1)		**16 (15)**	**1 (4)**	17 (13)	
	Others	23 (9.8)	4 (9.1)	27 (9.7)		13(13)	**5 (22)**	18 (14)	
	Shock/object/person	21 (8.9)	3 (6.8)	24 (8.6)		6 (6)	0 (0)	6 (5)	
Total		235 (100)	44 (100)	279 (100%)		104 (100)	23 (100)	127 (100)	

*M*, male; *F*, female; *n*, frequency

Source: Data obtained from the medical records in Hospital de Urgência de Teresina – PI, 2019 and 2020

In 2020, motorcycle accidents, for female (*n*=12; 52%) and male (*n*=42; 40%), were also the most frequent etiological cause. In male, physical aggression (16%) and falls (*n*=16; 15%) were the second and third most frequent causes, while in female, the second most frequent causes were other varied causes (22%).

Statistical analysis using the Pearson chi-square test, related to etiological factor and age, showed a proportionally significant association in 2019 between physical aggression and the age 30–39 (*x*² (32 =77.67; *p*=0.000; Table 3). Also, the etiological factor of falls and age between 50 and 100 years demonstrated a proportionally significant association, emphasizing an effect on the etiology of facial fractures (*x*² (32) =77.67; *p*= 0.000; Table 3). In 2020, no significance was observed between age and etiology (*x*² (28) = 3524; *p*=0.163; Table 3), due to the large reduction in the frequency of facial fractures of the population, causing discrepancies among the analyzed cohorts. The Fisher exact test did not show a significant association between sex and etiology in 2019 (Table 4).

### Severity

Descriptive statistics of the anatomical location of facial traumas showed a total of 772 injuries to the face, in which the main injuries were identified in the zygomatic complex, nasal region, jaw, and 364 cases of facial laceration. Furthermore, the data used to obtain the CFI score in relation to the year of occurrence of the trauma to determine the severity of the facial fracture is described in Table 5. Also, the main therapeutic interventions were osteosynthesis of the fractures and reduction with osteosynthesis in both years.

**Table 5:** Topographic distribution of facial injuries according to year of occurrence and therapy, Hospital de Urgência de Teresina - Teresina, Piauí, Brazil – 2019 and 2020

Anatomical location of the fracture for acquisition of the CFI score	Year of trauma occurrence		
	2019	2020	Total (*n* = frequency)
Dento-alveolar	0	1	(*n*) 1
	0.0%	100.0%	100.0%
Complex jaw fracture	**44**	**26**	(*n*) 70
	**62**.**9%**	**37**.**1%**	100.0%
Condyle	11	9	(*n*) 21
	**52**.**3%**	**47**.**61%**	100.0%
Le fort I	2	0	(*n*) 2
	100.0%	0.0%	100.0%
Le fort II	1	8	(*n*) 9
	**11**.**1%**	**88**.**9%**	100.0%
Le Fort III	1	1	(*n*) 2
	50.0%	50.0%	100.0%
Naso-orbito-ethmoid (NOE)	9	5	(*n*) 14
	64.3%	35.7%	100.0%
Maxillary zygomatic complex	104	35	(*n*) 139
	**74**.**8%**	**25**.**2%**	**100**.**0%**
Nasal	64	33	(*n*) 97
	**66**.**0%**	**34**.**0%**	**100**.**0%**
Orbital floor or rim	2	0	(*n*) 2
	100.0%	0.0%	100.0%
Angle	9	4	(*n*) 13
	**69**.**2%**	**30**.**8%**	100.0%
Symphysis	11	2	(*n*) 13
	84.6%	15.4%	100.0%
Complex fracture of the maxilla	19	4	(*n*) 23
	**82**.**6%**	**17**.**4%**	100.0%
Body	4	0	(*n*) 4
	100.0%	0.0%	100.0%
Laceration	265	99	(*n*) 364
	**72**.**8%**	**27**.**1%**	**100%**
Total	546	226	(*n*) 772
	70.7%	29.27%	100.0%
Therapy			
Surgical reduction with osteosynthesis	**64 (63%)**	**33 (34%)**	97
Surgical treatment for multiple trauma patients	23	14	37
Fracture osteosynthesis	**161 (71**.**6%)**	**64 (28**.**4%)**	225
Surgical treatment without osteosynthesis	20	10	30
Surgical reduction without osteosynthesis	11	6	17
Total	279	127	406

Source: Data obtained from the medical records in Hospital de Urgência de Teresina – PI, 2019 and 2020

The distribution of facial fractures according to the categories obtained through CFI scores related to the severity of injury indicated that in 2019, most part of the fractures presented a statistically significant proportion with moderate severity (*x*² (2) =19.56; *p*=0.000). However, fractures presented a statistically significant proportion with mild severity in 2020 (*x*² (2) =19.56; *p*=0.000), when compared to 2019 (Table 6).

**Table 6:** Descriptive and inferential statistics in relation to the CFI score according to age, type of fracture, sex, and etiology, Hospital de Urgência de Teresina - Teresina, Piauí, Brazil – 2019 and 2020

**Variable**	**Total**	**CFI score – 2019**	
	***N***	**Media**	**Value of** ***p***
Age	279	2.48	^(1)^*p* = 0.30
1 to 18	25	2.88	
19 to 29	105	2.45	
30 to 39	70	2.41	
40 to 49	43	2.37	
50 to 100	36	2.47	
Sex			^(1)^*p* = 0.68
M	235	2.48	
F	44	2.45	
Etiology			^*(1)^*p*=0.022
Motorcycle accident and other causes	110 e 27	2.69 e 1.96	
Type of fracture			
Maxillary/zygomatic bone and mandible fracture	132 e 74	2.62 e 2.62	^*(1)^p=0.001
Maxillary/zygomatic bone and multiple fractures involving skull bones e multiple fractures	132 e 7	2.62 e 3.0	^*(1)^*p*=0.016
Nasal bones fracture and mandible fracture	64 e 74	2.0 e 2.62	^*(1)^*p*=0.00
Nasal bones fracture and zygomatic/maxillary bone	64 e 132	2.0 e 2.62	^*(1)^*p*=0.00
Variable	**Total**	**CFI score - 2020**	
	***N***	**Average**	**Value of** ***p***
Age	127	2.44	^(1)^*p*=0.36
1 a 18	10	2.88	
19 a 29	41	2.21	
30 a 39	34	2.55	
40 a 49	25	2.60	
50 a 100	17	2.29	
Sex			^(1)^*p*=0.51
M	104	2.49	
F	23	2.21	
Etiology			
Physical aggression and motorcycle accident	19 e 54	1.84	^*(1)^*p*=0.006
Motorcycle accident and other causes	54 e 18	3.0	^*(1)^*p*=0.002
Type of fracture			
Zygomatic/maxillary bone fracture and nasal bones	55 e 33	2.95 e1.48	^*(1)^*p*=0.00
Nasal bones fractures and mandible	33 e 37	1.48 e 2.54	^*(1)^*p*=0.00

Kruskal-Wallis test ^(1);^ *Level of significance 5%; *N*, frequency; *CFI*, Comprehensive Facial Injury Score

Source: Data obtained from the medical records in Hospital de Urgência de Teresina – PI, 2019 and 2020

The average CFI score in 2019 was higher than in 2020 (2.48 against 2.44). The Kruskal-Wallis statistical test demonstrated that the type of fracture (*x*^2^ (4) =51.069; *p*=0.000) and etiology (*x*^2^ (8) =20.22; *p*=0.010) has an effect on the sum of the CFI score, in which the post-hoc pairwise comparisons showed that the zygomatic arch/maxillary fracture group differed statistically from mandible fractures (*p*=0.001) and multiple fractures involving the skull bones (*p*=0.016). In addition, fractures of the nasal bones differed statistically in relation to mandible fractures (*p*=0.00), the zygomatic and maxillary bone (0.0010), and the group of fractures caused by motorcycle accidents, differed statistically in relation to other causes of fractures.

Inferential statistical analysis using the Kruskal-Wallis test in the year 2020 also showed that physical aggression and motorcycle accidents occurred statistically differences in CFI scores (*x*^2^ (7) =28.61; *p*= 0.006), indicating that motorcycle accidents show a higher score. The Kruskal-Wallis test also identified that the type of fracture has an effect on the sum of the scores, in which the post-hoc comparisons indicated that the fractures of the nasal bones in relation to the zygomatic/maxillary bone differed statistically (*x*^2^ (3) =41.04; *p*=0.00). Also, fractures of the nasal bones differed statistically in the sum of the scores in relation to mandible fractures (*x*^2^ (3) =41.04; *p*=0.00), showing that the fractures, of the zygomatic bone and of the mandible, presented statistically greater confirmation. Statistical comparisons between the 2019 and 2020 cohorts, through comparisons by peers, between the most frequent fractures and etiology, show a significant effect on the sum of the CFI score (Table 6).

## Discussion

In this study, it was identified that the number of victims of facial fractures was lower in the prevailing period of social distancing by COVID-19, with a 54.5% reduction (*n*=279 in 2019 and *n*=127 in 2020), according to the literature [9, 11, 14]. This result was expected, as there was a 70% reduction in parks movement, 71% in commercial activities, 64% in leisure, and 34% reduction in mobility in relation to people who were unable to work in person [19].

The most affected age group by facial fractures was between 19 and 29 years, in both cohorts (2019 and 2020), with an average age of 34 and 28 years for men and women, which corroborates with the literature [14, 20]. This data may be the reason why individuals are more active in social, professional, and sporting environments at this age and are prone to facial fractures [15].

Facial fractures were more frequent in males in both cohorts, 84.2% in 2019 and 81.19% in 2020, corroborating with other authors [16, 21]. Activities performed by men are more likely to incidents, high-risk driving, interpersonal conflicts, and radical sports, leading to greater risks of facial fractures [7, 22].

The highest frequency of facial fractures was found in the urban zone, *n*=87.1% in 2019 and 90.6% in 2020, corroborating published data [7, 17]. This outcome may be explained by population aggregation in urban environment, predisposing to accidents and conflicts among individuals, contributing to the increased incidence of facial fractures [7, 23].

Motorcycle accident was the main etiological factor of the middle third fractures of the face, in both cohorts, 39.4% in 2019 and 42.5% in 2020, with a growing proportion in social isolation period by COVID-19, corroborating with other authors in relation to the great frequency of traffic accidents in developing countries [16, 24, 25].

The growth in the number of motorcycle accidents during the pandemic may be explained by the increase in online shopping, resulting in a greater distribution of motorized deliverymen in urban regions [26, 27]. However, our study identified a statistically significant association between physical aggression and age between 30 and 39 in 2019, in agreement with previous results that highlight more fractures by aggression in this age [28, 29]. In 2020, no statistical significance was observed between age groups and trauma etiologies (*x*² (28) = 35.24; *p*=0.163; Table 3), contradicting the study by Canzi et al. [30] in 2021 in Milan, in which there was an increase in the average age of trauma in 2020 during the Lockdown period. It is believed that these results can be explained by the large reduction in the frequency of facial fractures in the studied population, as it was an atypical period in society, causing discrepancies between the cohorts analyzed. Furthermore, differences in the social context, legislation, and culture in different regions of Brazil may present heterogeneous epidemiological data compared to international literature [1, 30].

In relation to the main etiological factor approached in contrast to other findings [17, 21, 31], they demonstrate that the main etiological factor of facial fractures was physical aggression. These facts may be explained by the differences in legislation, culture, economy, and politics of each region [22]. Moreover, a proportionately significant association was found (*p*<0.05) between falls and age from 50 to 100 in the 2019 cohort, demonstrating that the elderly are predisposed to falls [17]. In the 2020 cohort, there was an increase in the number of physical aggression, being the second most frequent cause during the COVID-19 pandemic [32].

Zygomatic bone fractures were the most frequent type of facial fractures in both years, 47.3% (2019) and 43.3% (2020), corroborating with studies in Brazil and other countries [14, 25]. The results showed the statistical significance of zygomatic bone fractures in relation to motorcycle accidents and physical aggression in the 2019 cohort (*p*<0.05). Also, in 2020, data demonstrated a statistically significant proportion of zygomatic bone in motorcycle accidents which is also reported by other authors [2, 14, 27]. Zygomatic bone is the side pillar of the face and absorbs a large part of the traumatic force in traffic accidents and physical aggressions, as they have a high kinetic load on impact [25].

Different from the results presented, other authors demonstrate a higher frequency of fractures, such as mandible and nasal bones ones, in which this research corresponded to the second and third types of fracture, in both years [31, 33]. The nasal bone is placed in the sagittal area of the face and has less bone thickness, making it less resistant to trauma impact [26]. The nasal bone tissue is susceptible to low-speed fractures or high kinetic load; in this case, it causes quadrangular cartilage fractures, so such factors contribute to the increased incidence of fractures in this area [27, 28].

It was observed that mandible fractures were mainly related to motorcycle accidents. The mandible is the only facial mobile bone and has an exacerbated prominence, being directly exposed in cases of facial trauma [28]. However, the results of Cohn et al. [31] and Juncar et al. [10] emphasize that mandible fractures are more frequent in victims of physical aggression. These differences may be explained by cultural, economic, and sociopolitical variations in the epidemiological pattern of fractures of each area [10].

The inferential statistical data in 2020, a period of social isolation due to COVID, highlight a statistically significant proportion of nasal fractures in physical aggressions and fractures by shock (*p*<0.05) compared to 2019 (*p*>0.05). The growth in the number of nasal fractures due to interpersonal violence, in this atypical period, may be related to social isolation, sociopolitical conflicts, economic stress, and family issues [17, 29, 34].

The severity of facial fractures was higher in the 2019 cohort (2.48—2019 versus 2.44—2020), in comparison to the COVID-19 pandemic period. In opposite to our findings, Ludwig et al. [32] demonstrated that the severity of traumatic injuries was higher during the social isolation period by COVID-19. These results must be carefully analyzed, because the difference in the proportion of fractures between the studied years is a factor which influences the statistical analysis of data. In addition, there are few publications in relation to the severity of injuries in a comparative form, before the pandemic and the period of social isolation; therefore, our data represent a promisor scientific and clinical impact for the community.

The main etiological factor before and during social isolation by COVID-19 were the motorcycle accidents, mainly in individuals from the urban zone. The frequency and severity of facial fractures were lower during the pandemic period. The main bone affected by facial fractures was the zygomatic in both years. Thus, this study may contribute to epidemiological knowledge and management of facial fractures in future situations and educational campaigns in traffic, for greater public awareness.

## Data Availability

Not applicable.
